# History of the American Ambulance, Established in Paris during the Siege of 1870-’71, Together with the Details of Its Methods and Its Work

**Published:** 1874-07-01

**Authors:** 


*From Jansen, McClurg & Co.*

HISTORY OF THE AMERICAN AMBULANCE, Established in Paris during the Siege of 1870–'71, together with the Details of its Methods and its Work. By Thomas W. Evans, M.D., D.D.S., Ph.D., President of the American International Sanitary Committee, Commander of the Legion of Honor, &c., &c. London: Sampson Low, Marston Low and Searle, 1873. pp. 694.

One proceeds to review a work, whose author is a Commander of the Legion of Honor, with something very like trepidation. The eminence of the writer's station forces upon the critic the necessity of looking upward, in order to comprehend the details of his work.

The book is, indeed, a great book. Its six hundred and ninety-four pages are perfectly printed in clear type, with wide margins; its title page is elaborately embellished with colored lettering; its ten plates and sixty-one wood-cuts admirably illustrate the skill of the engraver; in short, its perfection in every detail is such that the bibliophile might well regard it with a look of envy.

The subject-matter, however, presenting as it does much that is really instructive and valuable, might be profitably condensed into a book of much smaller dimensions. The stateliness of the text is generally in complete accordance with the impressiveness of the typography. There is rather a ludicrous contrast presented, however, between the passages which give in full the semi-diplomatic correspondence between the Queen of Prussia, the ex-Empress of France, and the President of the Commission, and the paragraphs in which we are informed that "soap and water, or molasses and water were generally employed for injections." Of the style of the work, it may be said, that while it is at times unexceptionable, there are numerous evidences of faults, which are common to those who transfer, instead of translate, words or ideas. For example, when we read (p. 564) of "the propriety of the cabinets," we know that the author means the cleanliness of the closets; but, as a matter of fact, he does not say it, either in the English or French language. Similar misuses of other words occur, as "installation" for "construction;" "degradation" for "soiling;" and such unnecessary gallicisms abound as, *connaissance de cause*, *cabinets d'aisance*, *en permanence*, &c.

The Surgical History of the Ambulance, is from the pen of Dr. John Swinburne, who takes occasion therein to charge an honored and esteemed veteran of our profession, Dr. Gurdon Buck, of New York, with "a very delicate kind of professional plagiarism." We venture to say that it will be long indeed, before the name of the latter is dissociated from the method of making extension in fractures, which he first employed in the New York City Hospital, and the name of the Surgeon-in-Chief of the American Ambulance substituted for it. Two hundred and forty-seven cases are reported in the Surgical History, and twenty-four in the Medical History, which is compiled by Dr. Wm. E. Johnston,—in all, two hundred and seventy-one patients were cared for by the organization.

The author solicits a generous criticism, in behalf of himself and his associates, while he admits that errors may be found in his book. Will he kindly forgive us if we say we can scarcely repress a smile, as there passes before us the procession which he conjures into view? Foremost of the line comes the Doctor of Medicine, Dental Surgery and Philosophy, officer and member of various orders, wearing upon his breast the grand cross of the order of St. Stanislas of Russia. Next come the Surgeon- and Physician-in-Chief, surrounded by their fifty-nine assistants, including the Marchioness of Bethisy. The two hundred and forty-seven surgical patients follow these, each carrying a photograph and wood-cut of his integumentary wound. To these succeed, at a distance, the awkward squad of twenty-four medical cases. Last of all approaches The Great Book, on stilts. We can almost see the inextricable confusion of the stars and stripes, and the red-cross flag. Hark! that is the "tintin" (English: rub-a-dub,) of the French drum!

And yet the author is not happy! This is, after all, only the first volume of a series which shall give the general history of Sanitary Associations, during the Franco-German war of 1870–'71!

"Tintin, tintin, r'lin, tintin."

How the gay procession clogs, for a brief hour, the dusty thoroughfares where we drive our homely gigs!

F. H. D.

---

MORTALITY table for Chicago from May 16 to May 30, 1874: Abscess of Liver 2, Apoplexy 5, Asphyxia 2, Asthma 1, Obstruction of Bowels 2, Disease of Brain 2, Compression of Brain 1, Congestion of Brain 1, Inflammation of Brain 8, Softening of Brain 3, Bronchitis 4, Cancer of Bladder 1, Cancer of Breast 1, Cancer of Pelvis 1, Cancer of Stomach 2, Cholera Infantum 3, Cholera Morbus 1, Consumption 34, Convulsions 41, Croup 5, Cyanosis 1, Cynanche Trachealis 1, Debility 4, Diarrhœa 2, Diphtheria 2, Dropsy 3, Dropsy of Chest, 1, Dropsy of Abdomen 2, Dysentery 2, Embolism 1, Enteritis 17, Epilepsy 30, Erysipelas 6, Puerperal Fever 3, Remittent Fever 1, Scarlet Fever 2, Typhoid Fever 4, Gastritis 6, Gastro Enteritis 2, Disease of Heart 9, Fatty Degeneration of Heart 1, Hernia 2, Hydrocephalus 9, Inanition 7, Intemperance 1, Bright's Disease of Kidneys 6, Disease of Liver 1, Congestion of Lungs 8, Hemorrhage of Lungs 1, Disease of Lungs 2, Malformation 1, Measles 1, Meningitis 8, Cerebro-spinal Meningitis 2, Tubercular Meningitis 1, Myelitis 1, Metritis 1, Œdema Pulmonum 1, Old Age 5, Paralysis 3, Peritonitis 4, Pleurisy 2, Pneumonia 13, Typhoid Pneumonia 1, Pericarditis 5, Pyemia 2, Septicemia 1, Small-pox 10, Disease of Spine 1, Ulceration of Stomach 1, Tabes Mesenterica 10, Teething 2, Whooping Cough 6, Accidents 13, Suicide 5. Total, 336.

